# Comparison of perception of stress and consumption of anxiolytics in hospital and out-hospital conditions: a cross-sectional study

**DOI:** 10.3389/fpubh.2024.1339246

**Published:** 2024-02-19

**Authors:** Željko Jovanović, Sara Pešut, Bojan Miletić

**Affiliations:** ^1^Faculty of Health Studies, University of Rijeka, Rijeka, Croatia; ^2^Faculty of Medicine, Josip Juraj Strossmayer University of Osijek, Osijek, Croatia

**Keywords:** anxiolytics, benzodiazepines, stress, work, healthcare professional

## Abstract

**Background:**

The workplace is a place where medical workers are exposed to extreme stress, particularly during medical emergencies or events of epidemic or pandemic proportions. Anxiolytic therapy is often used to overcome professional challenges. Deepening knowledge about the prevalence of the use of anxiolytics and the perception of stress among medical workers enables the timely recognition of problems and the preparation of measures to improve the working conditions and quality of life of medical workers. The study’s primary objective was to investigate whether there were differences in the usage of anxiolytics among healthcare professionals in and out of the hospital. In addition to the main objective, there are other objectives that have been established: To examine whether there are statistically justified differences in stress perceptions between hospital and outpatient healthcare professionals; 2. To examine the stress factors in the workplace in both hospital and outpatient settings. To compare the frequency of taking anxiolytics with respect to various variables (age, seniority, occupation and level of education); 4. determines the impact of working conditions on stress perception and life satisfaction in healthcare professionals. The design of research: Cross-sectional research.

**Materials and methods:**

The research involved 159 healthcare professionals in Slavonski Brod: 96 employees of the General Hospital “Dr. Josip Benčević” and 63 employees of the Health Center and the Institute for Emergency Medicine of Brodsko-Posavina County. Respondents were able to participate in the study by filling out questionnaires online. The questionnaire was designed to be voluntary and anonymous and contained 53 questions.

**Results:**

Statistically significant differences were shown in the perception of stress, which is greater in hospital staff, than in the difference between stressors in the workplace, where hospital staff showed higher values in all categories, but three factors are more significant differences: “Organization of the workplace and financial issues,” “Conflicts and communication at work” and “Professional and intellectual requirements.” There are significant differences in the frequency of using anxiolytics with the assistance of a psychiatrist. Working conditions have a much greater impact on the perception of stress and life satisfaction in hospital staff, while in hospital staff only a weak link between the perception of stress and life satisfaction is expressed. Anxiolytics are consumed by 27.10% of hospital workers and 23.80% of outside-the-hospital workers.

**Conclusion:**

The consumption of anxiolytic drugs by healthcare professionals in hospital and outpatient conditions does not make a significant difference, but they do have statistically significant differences in their perception of stress.

## Introduction

The social and economic development of society is greatly influenced by mental health, which is a fundamental right of every individual. One of the world’s top public health priorities is promoting mental health (1). The Fourth European Working Conditions Survey of 2005 indicated that work stress is among the most common health problems related to work and affects 22% of workers (2). According to the 2020 EU labour force survey, 44.6% of employees report encountering risk factors at work that negatively impact their mental health (3). Stress is defined as a discrepancy in the psychophysical balance of an individual that occurs when the demands of the environment go beyond the possibilities of solving and coping with problems, which is why there is a feeling of danger (3, 4). Health care professionals are a profession highly exposed to stress due to great responsibility for human health and lives, constant threats of lawsuits from dissatisfied patients, excessive workload, dissatisfaction in the workplace and/or in the family. Stress is contributed by a busy work schedule and staff shortages, which is why many work overtime, also shift and night work, on-call, emergencies, care for the seriously ill and dying, conflicts at work, etc. Research indicates that health care workers, under the influence of stress, are more likely to succumb to poor diet, smoking, alcohol, and drugs (5–10). Although there are many similarities, stress and stress factors are not the same in hospital and outpatient conditions (8). In examining the sources of professional stress of nurses in family medicine clinics conducted in 20 outpatient clinics of the Health Center in the city of Zagreb, the most important stressor was the burden on the scope of work and time pressures, which according to the authors is a characteristic phenomenon associated with the work of nurses in family medicine clinics (9). Similar results are provided by research on occupational stress, satisfaction and burnout of nurses and technicians employed in the field of palliative care (10). In a study of 459 doctors of the Clinical Hospital Center Rijeka, emotional exhaustion of a high degree was recorded in 43.6%, and it is concluded that stress at work can have a negative impact on mental health and the quality of work of doctors, so it is necessary to take targeted measures for the prevention and reduction of stressful events (11). Also, research on stress in health care professionals in hospitals in Zagreb shows that the biggest stressors in both doctors and nurses / technicians are financial and organizational factors, and in nurses additionally fear of specific dangers associated with workplaces in healthcare (12). In a state of stress, it is possible to work for a while without major consequences, but everyday stress overloads the body, which is reflected in the mental and physical ability of employees. Accordingly, there is dissatisfaction, exhaustion, reduced productivity, and quality of the work done, and potentially a greater number of mistakes that can be fatal in the health profession, as well as professional injuries at work and the consequent greater absence from work (13, 14). To make it easier to endure everyday stressful situations, many people, including health professionals, reach for some form of anxiolytic therapy (8). Anxiolytics are drugs that help relieve or eliminate feelings of tension, anxiety, fear, restlessness, and irritability without leading to stronger fatigue than hypnotics (15–17). In Croatia, we monitor a steady growth in consumption of 1.58% per year from 2016 to 2020. Anxiolytic consumption amounts to HRK 91 million per year. During 2020, the consumption of anxiolytics belonged mostly to benzodiazepines, especially diazepam and alprazolam, taken every day by 81 people out of 1000 (16). According to HALMED from 2016 to 2020, Brod-Posavina County was eighth in terms of anxiolytic consumption in 2020, the first being Koprivnica-Križevci County and the last being Istria County (16). Although more research is available in Croatia dealing with stress in health care workers and the impact of stress on personal life, few of them have dealt with the frequency of anxiolytics. One Brazilian study, based on published studies from 2008 to 2017, analyzed the evidence on the factors and implications associated with the use of psychoactive substances in healthcare professionals (8). The factors that led to such a condition were working environment conditions, physical and psychological exhaustion, long working hours, and easy access and handling of medications. As the main conclusion of these authors, it is emphasized that the working environment is the main factor and predisposing factor for the use of psychoactive substances in medics. The drugs with the highest consumption were sedatives, morphine, antidepressants, barbiturates, analgesics, amphetamines, and benzodiazepines because they are more accessible within health care facilities (8). It is unquestionable that stress in healthcare professionals is a global problem and that much more research needs to be devoted to it, and equally approaching better coping and relieving stress or removing stressors from the work environment. The main objective of this study was to examine the differences in the perception of stress and the use of anxiolytics in hospital and outpatient workers. In addition to the main goal, specific goals were determined: to compare the stress factors of health workers in the workplace in hospital and outpatient conditions, to compare the frequency of taking anxiolytics, and to determine the impact of working conditions on stress perception and life satisfaction. These goals led to the creation of a null hypothesis, which assumes that there is no statistically significant difference between the groups being examined.

## Materials and methods

### Study design and participants

The research took place as an academic (non-commercial) observational, IV phase, and cross-sectional study, with 159 participants, nurses/technicians, doctors, and other health workers from Slavonski Brod. The General Hospital “Dr. Josip Ben eve” interviewed 96 respondents to survey the hospital environment, and the Health Center and the Institute interviewed 63 respondents.

Samples have been taken by the emergency medicine department of Brod-Posavina County for the outpatient environment. Males made up 20.8% of the total sample, while females made up 79.2%, or 126 people. The research, approved by the Ethics Committee of the General Hospital “Dr. Josip Benčević,” with the consent of the Health Center and the Institute of Emergency Medicine, was conducted in April and May 2022. The STROBE cross-sectional reporting guidelines were employed in the preparation of the study. To conduct the research, Google Forms were used to create an online survey questionnaire that was accessed by the respondents by going to the link on Google Drive. The questionnaire was shared online by the employees of the mentioned institutions who took part in the survey. The questionnaire had a total of 53 questions, which were divided into 4 parts. The questionnaire’s initial segment called ‘Sociodemographic Data’ comprised 10 questions meant to be used in research. This section collects sociodemographic data including gender, age, marital status, household, level of education, occupation, workplace, length of service, working hours, and type of employment. The authors created the second questionnaire section, ‘Anxiolytics (Tranquilizers),’ which had 12 questions. The initial question dealt with the general satisfaction of life. It was necessary to mark the number that best describes the life satisfaction on a Likert scale of 1 to 10, where 1 is not at all satisfied and 10 is completely satisfied. The other 11 questions related to the frequency, causes and consequences of taking anxiolytics, and the impact of the working environment and the COVID-19 pandemic on the need to use anxiolytics. Most were multiple choice questions, except for two open-ended questions, in which those subjects who stated that they were using anxiolytics could state the main reason for taking them, and the question about side effects of anxiolytics. A rating is given to the frequency of taking based on the scale of choice, which ranges from never, rarely, occasionally, and regularly. Also, this part of the questionnaire wanted to find out whether those who take anxiolytics do so during working hours and how it affects the quality of their work and performance of work obligations. The Perceived Stress Scale (Sheldon Cohen, 1994) is the third component and establishes the level of stress that health workers encounter in hospital and outpatient work settings. This part of the questionnaire consisted of 10 questions about the feelings and thoughts that the respondents had in the last month, and who evaluated their experience and frequency of stress according to the Likert scale with scores from 1 to 5, where: 1 – never, 2 – almost never, 3 – sometimes, 4 – quite often, 5 – very common. The score range for all answers is from 0 to 4, with the ‘never’ answers receiving 0 points and the ‘very often’ answers receiving 4 points. According to the instruction of the author Sheldon Cohen, answers 4, 5, 7, and 8 are inverted points, meaning they scored in reverse due to positively worded questions. All points were summed up, and a higher tally meant that respondents had a harder time perceiving stress in life. The consistency of the test was verified by the Cronbach alpha coefficient, which was 0.8941. This indicates very high reliability of the data. The last part of the questionnaire “Questionnaire of stressful conditions in the workplace of health professionals” was taken with the permission of the author from the 2010 questionnaire Milan Milošević, with minimal changes (the number of questions related to hazards at work, radiation, inhalation anesthetics, cytostatic was reduced, because the purpose of this survey is to assess the stress of hospital and outpatient health workers - the original work is concentrated only on the hospital part) (18). The issues are divided into six factor groups: (F1) “Workplace Organization and Financial Matters,” (F2) “Public Criticism and Court Action,” (F3) “Dangers and Harms at Work,” (F4) “Conflicts and Communication at Work,” (F5) “Shift Work” and (F6) “Professional and Intellectual Claims.” In addition to the questions from the six mentioned factor groups, at the end of the survey, the questionnaire contains another question, at the same time repeated, from the second part of the questionnaire, a question about general life satisfaction, which was intended to test the impact of filling out the questionnaire on the assessment of life satisfaction.

### Data analysis

Representativeness of the sample was ensured by the quota sampling method, which was based on professional qualifications and place of work, but the selection of respondents within the set quota was at random. To ensure the reliability of the survey, the sample size was calculated using an online calculator (https://www.questionpro.com/sample-size-calculator/oli MEDcalc) with a default confidence level of 92.5% and an error limit of 5%. The minimum response rate was determined to be 151 people. Due to the usual turnout rate of 70%, the number of surveys conducted increased to 200 people, 159 were collected, systematized, and processed and entered into the database for further statistical analysis. MedCalc® Statistical Software version 19.6 was utilized for the statistical program. Descriptive statistics are used to describe the data for each variable and scale. The absolute and relative frequency of all variables (categorical and numerical) is used to represent them. The central tendencies for categorical variables are represented by mode, while numerical variables are represented by the arithmetic mean and standard deviation as an indicator of dispersion. The Shapiro–Wilk test was used to analyze the normality of the distribution of numerical data, while the Lewen test was used to test for homogeneity. Categorical variable analyses were performed by Fisher’s exact test, Pearson’s Chi-square test (*χ*^2^ test) and Wilcoxon’s test, while numerical variables were analyzed by Kruskal-Walis nonparametric test and one-way variance analysis. The post-hoc tests used to test the significance of differences are the Scheffle test for parametric analyses and the Bonferroni z-test for nonparametric analyses. The Cronbach alpha test validated the validity of the results for stress perception tests and stressful work conditions. Logistic regression determined the probability of predicting events based on nominal variables. Variable connectivity strength was tested using Spearman’s rank correlations. The significance level for all analyses carried out is set to Alpha = 0.05.

### Ethical consideration

The research was conducted in accordance with the ethical rules and with the permission of the ethics committee of the institutions where the research was conducted, in compliance with the Personal Data Protection Act in Croatia (Official Gazette 103/03–106/12) and in accordance with the rules of the Declaration of Helsinki. The research required each respondent to be aware of the rules and necessary information. Participation in the research was completely voluntary. Confidentiality is maintained in the processing of all data, and the anonymity of the obtained data is guaranteed.

## Results

The survey data is systematized and described in tables. The respondents had an average age of 41.35 years. Most respondents are married and live in a household as a family with children. The most common form of education is graduate studies. The largest number of health professionals are employed in the hospital, and the average tenure is 17.67 years. Most people are employed full-time. Table 1 shows descriptive statistics of all answers to the questions on the Stress Perception Scales. Before testing the first target, the normality of distributions of all numerical variables was checked by the Shapiro–Wilk test, and the homogeneity of the data was verified by the Levene test. To determine the reliability of the measurement scales of the “Stress Perception” and “Stress Factors” tests and the consistency of the response, Cronbach alpha (*α*) coefficients were calculated. The Cronbach alpha coefficient is a measurement of the consistency of a set of statements and can be between 0 and 1. The closer the value of 1 is, the more consistent the claims are with each other. The achieved value of the α- coefficient for the test “Stress Perception” was *α* = 0.89, and for the test “Stress Factors in the Workplace,” which is grouped into six factors, it was *α* = 0.87. The Cronbach alpha coefficient results indicate that the scales applied are highly reliable. The results obtained from the questionnaire are abundant and complex, and the detailed statistical analysis is summarized and focused only on the specific goals of this research set at the beginning of the paper.

**Table 1:** Descriptive statistics of answers to the questions “Stress at the workplace.”

**Factor**	**Variable**	**N**	**Arithmetic mean**	**SD**	**Median**	**Quartiles Q1 – Q3**
F1: Workplace organization and financial issues	Inadequate personal income	159	3.11	1.26	3	2 to 4
	Inadequate workspace	159	3.11	1.33	3	2 to 4
	Low chance od advancement	159	2.81	1.30	3	2 to 4
	Insuficient number of employes	159	3.59	1.27	4	3 to 5
	Poor organization of work	159	3.38	1.27	3	3 to 4.75
	Everyday unforseen situations	159	3.26	1.09	3	3 to 4
	Administrative affairs	159	3.23	1.32	3	2 to 4
	Work overload	159	3.75	1.13	4	3 to 5
F2: Public criticism and court lawsuits	The threat of lawsuit	159	3.13	1.50	3	2 to 5
	Conflicts with patients	159	3.03	1.36	3	2 to 4
	Non-seoaration of professional and private life	159	2.93	1.26	3	2 to 4
F3: Hazards and harms at work	Dealing with incurable patients	159	3.47	1.16	3	3 to 4
	Fear of exposure to harmful agents in the work environment	159	2.93	1.32	3	2 to 4
F4: Conflicts and communication at work	Conflicts with colleagues or superiors	159	3.20	1.33	3	2 to 4
F6: Professional and intellectual requirements	Bombardment with new information	159	2.99	1.17	3	2 to 4
	Timeline pressure	159	3.19	1.15	3	2 to 4
F5: Shift work	Shift work	159	2.64	1.39	3	1 to 4
	Regular work	159	2.50	1.16	3	1.25 to 3
	Work in rot	159	2.62	1.38	3	1 to 3.75
	24-h standby	159	2.89	1.60	3	1 to 4

### Perception and stress factors at work

Table 2 provides the results of the variance analysis, which found that hospital and outpatient health care professionals differ significantly for factor F1, F4 and F6 at the level of significance (*p* < 0.05), while the differences in factors F2, F3, and F5 are random and not significant at the statistical level (*p* > 0.05), which also applies to overall stress levels and stress perception. Statistically, hospital and outpatient health professionals experience stress at work in significant differences. Of the 159 respondents, 84 in hospital conditions said their job was stressful, while the expected number of positive responses was 77.3, which is more than 5% of respondents than expected. Likewise, 6.3 less people responded from the outpatient system that their workplace was more stressful than expected. Compared to expectations, 5.9 subjects from the hospital reported fewer negative emotions about stress at work, while 5.9 more subjects from the outpatient system reported negative feelings about stress at work. Based on the z-test (according to the Bonferroni method), it was found that there are significant statistical differences between workplace stress in a hospital versus outpatient service, as well as for those people who consider the workplace not to be stressful outside the hospital or in the hospital.

**Table 2:** Results of variance analysis of stress factors in the hospital and outside the hospital.

	*F*	*P*
**F1 - Workplace organization and financial issues**	25,150	**0,001**
F2 - Public criticism and court lawsuits	1,831	0,178
F3 - Hazards and harms at work	2,646	0,106
**F4 - Conflicts and communication at work**	9,467	**0,002**
F5 - Shift work	0,750	0,388
**F6 - Professional and intellectual requirements**	24,327	**0,001**
**Total stress levels in the workplace**	18,36	**0,001**
**Perception of stress**	0,165	0,685
*F < 3,25 = H_0_ *F* > 3,25 = H_1_		

### Frequency of taking anxiolytics

The frequency of taking anxiolytics regarding work in hospital and outpatient conditions and the overall impact of stress and stress conditions were processed with Pearson’s Chi-square test and the value *χ*^2^ = 0.212 was calculated, which was at the level of significance *p* = 0.645, i.e., there is no statistically significant difference between health workers in hospital and outpatient conditions. Additionally, the findings demonstrate that the workplace of health workers was not associated with the use of anxiolytics due to stress. Afterward, 41 individuals were examined, who gave positive responses to the question about the use of anxiolytics. As a small number of respondents responded positively to the question of the use of anxiolytics, it was necessary to combine the answers “sometimes,” “often” and “very often” into a common feature “often.” The chi-square test determined the value *χ*^2^ = 0.833 at the significance level of *p* = 0.361, which means that there is no difference in the frequency of anxiolytic use between healthcare workers in hospital and outpatient workplaces (Table 3). Furthermore, based on a sample of 41 subjects who confirmed that they were taking anxiolytics, the aim was to examine whether the frequency of taking anxiolytics regarding the occupation of health care workers, when there is already no link between frequency and place of work. Fisher’s exact test yielded a value of 1.0 with a value of *p* = 0.570, which is higher than the default limit value of 0.05, so no relationship between variable frequency of use of anxiolytics and occupations was proven. Statistical analysis of Variables by Kruskal-Walli’s analysis, where seniority was used as a numerical variable, resulted in a test value of 0.249, with *p* = 0.617, therefore there is no significant statistical relationship between seniority and the application of anxiolytics. As the final variable in this goal, the frequency of health workers taking anxiolytics with the help of a psychiatrist was observed. Pearson’s Chi-square test was used, and the results obtained *χ*^2^ = 25,278 with a level of significance *p* < 0.0001 show that variables are the help of psychiatrists and the application of anxiolytics in interrelated. The greatest contribution to statistical significance is made by people who have sought the help of a psychiatrist, i.e., people who have sought help but do not use anxiolytics. The frequency of anxiolytic use was also examined, and 41 people answered in the affirmative to the question about the use of anxiolytics. Pearson’s Chi-squared test with the value *χ*^2^ = 4.985; *p* = 0.0256 says that the variables “psychiatrist’s help” and “frequency of administration” (rarely and frequently) are related to each other. The next variant concerns the frequency of anxiolytic use in healthcare workers as compared to overall stressful working conditions and stress perception. The variance analysis for the variable ‘total stress impact and working conditions’ between people who used anxiolytics and those who did not found no significant statistical difference (*F* = 0.143 with *p* = 0.707). Conversely, in the analysis of variance for the variable “stress perception” between the two subpopulations (people who use and non-anxiolytics), significant statistical differences were found, *F* = 7.330 at the level of significance *p* = 0.001. Although this is not necessary, the post has Scheffe test was used to further test the significance of difference and clearly determined the significant differences between the two groups of subjects for stress perception (Table 4). People who rarely use anxiolytics scored on average 17, while those who frequently use anxiolytics scored significantly higher on average, on average, 23 points.

**Table 3:** Results of frequency of use of anxiolytics and workplace of healthcare workers.

		Frequency of use of anxiolytics		Total
		Rarely	Often	
Place of work	Hospital	10	16	26
	Out of hospital	8	7	15
Total		18	23	41
*χ* ^2^		0,833		
DF		1		
Significance level		*P* = 0,3613		

**Table 4:** Results of *post hoc* Scheffe’s test of factors F1, F4, F6 and total stress in working conditions between health professionals in the hospital and outside the hospital.

	Place of work	*N*	A. S.	S. D.
F1 (Workplace organization and financial issues)	Hospital	96	63,72	20,19
	Out of hospital	63	46,73	21,88
F4 (Conflicts and communication at work)	Hospital	96	61,46	29,00
	Out of hospital	63	45,24	37,26
F6 (Professional and intellectual requirements)	Hospital	96	49,54	25,64
	Out of hospital	63	29,56	23,95
Total	Hospital	96	57,59	18,98
	Out of hospital	63	43,95	20,60

### Correlation between working conditions and perception of stress and quality of life

The association of working conditions with the perception of stress and quality of life, and life satisfaction of health care workers in hospital and outpatient conditions were tested by Spearman rank correlation (Table 5) and there are differences between life satisfaction and the impact of working conditions on stress, as well as on the perception of stress between hospital and outpatient staff. Within the hospital staff, all links were statistically significant, except for the “Hazards and Harms at Work” factor. The connections between life satisfaction, working conditions, and stress perception were all negative. Life satisfaction and stress perception have been found to have a medium-strong negative correlation. The variables “Shift Work” and “Professional and Intellectual Requirements” had weak connections, while all other connections were very weak or insignificant and (Table 6) finally provides logistic regression of the possibility of assessing the appearance of factors influencing the use of anxiolytics.

**Table 5:** Spearman rank correlation of life satisfaction and factors influencing working conditions on stress and stress perception.

		**Hospital**	**Out of hospital**
F1 - Workplace organization and financial issues	Correlation *p*-value Total	−0,277 0,0063 96	−0,211 0,0975 63
F2 - Public criticism and court lawsuits	Correlation *P*-value Total	−0,248 0,0147 96	−0,243 0,0552 63
F3 - Hazards and harms at work	Correlation *P*-value Total	−0,180 0,0786 96	−0,098 0,4469 63
F4 - Conflicts and communication at work	Correlation *P*-value Total	−0,274 0,0070 96	−0,150 0,2418 63
F5 - Shift work	Correlation *P*-value Total	−0,367 0,0002 96	−0,209 0,0998 63
F6 - Professional and intellectual requirements	Correlation *P*-value Total	-0,314 0,0019 96	-0,031 0,8104 63
***Stress perception***	Correlation *P*-value Total	-0,709 < 0,0001 96	-0,490 < 0,0001 63

## Discussion

The topic of workplace stress among healthcare professionals is being widely discussed. In the health profession, there are specific stressors that are characteristic of healthcare. Stress in the workplace is often caused by shift work, a high level of responsibility, disrupted interpersonal relationships, and the anxiety disorders have increased significantly recently, mainly due to the profound transformations that occurred in the economic and cultural context that were accompanied by the pressures of a modern, technological and competitive society. (3–5, 14, 18). Specific stressors related to working conditions in a particular occupation occur, as well as those related to the ways of performing a particular job. When viewed within the framework of health professions, it is clear that there are a lot of specific stressors, from the fact that health professionals have a responsibility to someone’s life, through various risks to which they can be exposed, to working conditions and workers’ rights, such as the peculiarities of shift work, night work, emotional exhaustion, etc., for which a series of studies on health professionals in a number of countries from Spain and Brazil, until China and Ethiopia or Three Balkan Countries showed that they have an essential role in a greater propensity for the negative consequences of stress (6–9, 19, 20). In addition, health professionals are a group that is at increased risk of burn-out syndrome, i.e., burnout syndrome, mainly because they work intensively with other people, colleagues, patients, and the public (5, 19, 20). According to research, stressors in hospital and outpatient conditions are not the same, but long-term stress in health workers leads to post-traumatic stress disorder. Financial motives, as well as social recognition for responsible work in health care are an important factor of satisfaction, so nurses in a Chinese survey from 2009 particularly pointed out the poor image of nursing in Chinese society as a cause of stress, and organizational problems of work and the disparity between what they invest and receive, and in 2016 a similar Croatian survey conducted at the Clinical hospital “Dubrava” in Zagreb shows that nurses and technicians in the Republic of Croatia as the main stressor cite workplace organization and financial issues (19, 20). However, the main difference in the hospital and outpatient system relates to excessive burdens on administrative tasks, the number of patients and the time frame of fulfillment of these obligations in outpatient activities. Inadequate evaluation of the well-done, responsible, and very complex work of protecting the health of citizens is a major stressor for all health professionals. According to studies conducted among health professionals, particularly nurses and technicians, there is a significant relationship between certain illnesses and stress in the workplace. As for mental health problems, employees in the healthcare system may develop anxiety, depression, or insomnia, for which they may use various psychopharmaceuticals (21–23). In some countries, mental disorders associated with stress at work are represented among as many as 75% of employees, which accounts for more than two-thirds of patients, and of particular concern the so-called social stress, refers to the impact of the social situation in some period, where the health system leads (24, 25). Our goal was to determine if there is a difference in perception of stress and taking anxiolytics among healthcare professionals in both hospital and outpatient conditions to prevent stress condition and create potential intervention for future. The results show that nurses and doctors do not differ in the frequency of taking anxiolytics, but that there are significant differences in the perception of stress in employees in hospital and outpatient conditions. Based on the results obtained, it can also be concluded that stress in the workplace and the place of work are interrelated and that the place of work statistically significantly affects the stress of health workers. All this is important in an international environment in order to improve the working environment of health workers around the world. All employees who reported feeling stress in the workplace, a total of 128 out of 159, spoke about the relationship between stress and the workplace. Out of the 128 individuals who responded positively to stress in the workplace, 65.60% were workers in the hospital, while 34.40% were from outside the hospital. Among other things, 72.20% of workers in outpatient conditions have confirmed that they do not feel stressed while at work. Such results can be attributed to the content and manner of organizing work in the hospital and outpatient conditions. Although there is no statistically significant difference, employees in the hospital showed a higher level of stress in all stressors than in the Health Center and in the Institute of Emergency Medicine. The biggest difference is due to the first stressor, namely workplace organization and financial issues. This is precisely the reason for the frequent abandonment of the health profession, especially the nursing profession around the world. Similarly, display the previously mentioned research from all parts of the world, including some Croatian research (8–12). Hospital employees indicated this as the biggest stressor, and the least rated factor was “Professional and intellectual requirements,” which indicates the high quality of education and the level of competences of health workers in Croatia. At the same time, employees outside the hospital rated this factor as the least stressful, and as the highest rated stressors, outpatient staff these are “Dangers and harms at work” and “Shift work.” It can be observed that the level of stress experienced by workers in hospital conditions is statistically significantly higher. Also, it has been shown that there is no statistically significant difference in the perception of stress between nurses and doctors, which could also mean that clear boundaries between the roles of these professions are erased, precisely because of the requirements and shortcomings of the system, and that the same number of large obligations, administration and responsibilities is placed on all employees in the health care system. Also, this is one of the more common reasons why nurses and doctors leave Eastern European countries to work in other more developed countries of the European Union. In the outpatient segment, administrative tasks are a source of stress and dissatisfaction for employees. The peculiarities and differences in the frequency of use of anxiolytics between hospital and outpatient staff in this paper has a statistically small difference, which indicates that the workplace of health care workers in this study has nothing to do with taking anxiolytics due to stress, even though in the perception of stress these two groups differ significantly. It could be expected that the use of anxiolytics will increase since the values of stress perception are higher in hospital staff. Such results can be explained by some of the responses of the respondents, who showed that a part of the respondents who answered affirmatively about stress in the workplace, did not seek professional help in the form of psychiatric / psychological help. Although it is a health care staff, the social stigma toward mental disorders and disorders is still very strong in our country and many health professionals, although they feel a high level of stress, resort to self-help, which is often reflected as neglecting their own psychological state and trying to further cope with stress from day to day. Continuously educating health professionals and the public on the importance of promoting mental health from the youngest to retirement age is essential. Treating mental states and disorders as a disease is not a disqualifying factor, but rather as a disease that can and should be prevented and treated. The slight difference in the use of anxiolytics among hospital and outpatient staff is also because, given the responsibility and the number of daily demands and challenges, any health occupation is equally exposed to stress. As previously mentioned, the boundaries between the individual roles and tasks of health professions are being blurred by the increasing temporal and quantitative requirements. Being a nurse today means taking over part of the work of a doctor, but also vice versa due to the lack of nursing staff, although experience from intensive care units where highly educated and motivated nurses work shows possible positive solutions (26). In addition, on the example of health workers in Croatia, it can be concluded that the experience of stress and the use of anxiolytics are not related to the extent that could be expected before verifiable statistical measurements. Of the 159, 41 responded that they were taking anxiolytics, with a significance level of 0.3613, meaning that the null hypothesis is not rejected, and that here too there is a statistically small difference in the use of anxiolytics between workers in the hospital and outside the hospital. The reason for this lies in the fact that many health professionals clearly feel that they can cope with everyday stress without the use of anxiolytics. To achieve this goal, it would be beneficial to investigate the causes of the onset of anxiolytics in 41 individuals who have confirmed their use. This would more accurately link the working conditions and the place of work with their application, i.e., how many of them started taking anxiolytics due to stressors at work or possibly the cause was outside of work. Health professionals, regardless of their profession, have the same ability to perceive their ailments. Although seniority did not have a significant impact on the frequency of taking anxiolytics, it was found that individuals with longer work experience still take anxiolytics slightly more frequently. This link between seniority and age can also be explained by burnout syndrome at work. Burnout occurs after a long period of time working in one profession (27). In a study of the interrelationship between the use of anxiolytics and the seeking of psychiatric assistance, Fisher’s exact test showed statistically significant differences. As many as 80% of respondents said that they use anxiolytics with the help of a psychiatrist, and only 20% of them that they have sought the help of a psychiatrist, but do not apply anxiolytics. However, it would be more significant to examine the reasons why people who have sought the help of a psychiatrist do not use anxiolytics, whether it may be the use of some other method of treatment, psychotherapy or delaying pharmacotherapy. It was also observed that the use of anxiolytics and the help of a psychiatrist are related to each other, since among the 41 subjects who use anxiolytics, and they sought the help of a psychiatrist, there were 12 who use anxiolytics with the help of a psychiatrist, and 29 of them who use them without the help of a psychiatrist. Such results are somewhat worrying and are in favor of numerous studies already conducted on this topic, according to which health care workers are prone to addictions to anxiolytics because they are easily accessible and are not controlled when taking them, and often take them on their own (8, 27–29). The interdependence of the frequency of taking anxiolytics and the overall experience of stress showed a statistically significant difference, i.e., that people who use anxiolytics have on average more points on the stress perception test than those who do not apply them. Such a result correlates with a result that has shown that greater perception of stress also contributes to the more frequent use of anxiolytics, because it was found that there are differences in the perception of stress between people who use and do not use anxiolytics. When assessing the impact of working conditions on stress perception and life satisfaction, hospital staff have links between life satisfaction and working conditions, as well as the connection between life satisfaction and stress perception. In outpatient staff, there is no relationship between variables life satisfaction and working conditions. The relationship between stress perception and life satisfaction was weak for outpatient staff. The increase in stress levels of hospital staff led to a decrease in life satisfaction. In addition, due to their factors, the questions in the questionnaire were not sufficiently adapted to outpatient staff, i.e., working conditions, which were more based on the working conditions of hospital staff, so that outpatient staff could not get a statistically significant result on the real impact of working conditions on life satisfaction and stress perception, which is a lack of questionnaires and should be kept in mind for future research. Overall, it is interesting that after filling out the questionnaire, it turned out that the health care staff in total showed a change in attitudes about life satisfaction, and to the negative side, but within this number there are more hospital staff who showed negative changes. While the small number of subjects may be disadvantageous, as a pilot study, it holds great significance. To obtain more significant results and a larger sample, it is possible to combine these tests at both the state and regional level. To prevent the use or at least the frequency of the use of anxiolytics, it is inevitable, on the example of health workers, that work must be done to reduce stress conditions, which affect the perception of stress, and ultimately life satisfaction. The reduction of stressors in the working conditions of health workers can be achieved through system reform, continuous education, better distribution of shifts, improvement of material and other working conditions, increased days off or vacation days and similar refinement of both working and leisure time. In addition, too much orientation to work and occupation affect both private life and the complete picture of life, which can be changed by working on yourself, personal development and coping techniques when anxiolytics are not needed. The support of psychologists in the work environment in Croatia is more an exception than a rule. In spite of the aforementioned shortcomings of the research, although this is a small local study, it provides enough guidelines for ensuring greater job satisfaction of all employees in the healthcare system in the world, which will improve the health of the entire population worldwide, because the prerequisite for good health care is satisfied and healthy healthcare workers (30, 31). Considering the amount of data obtained, statistical analyses are summarized, and it could continue to examine the correlation and significance of each variable based on many other goals that are currently not in the focus of this paper but represent a good guideline for continuing research on a sample of the total population of employees in the health care system. All of this has additionally proved indispensable in the working conditions of health workers during the COVID-19 pandemic (26, 32).

## Conclusion

Hospital and outpatient health care workers in our study have no statistically significant differences in their use of anxiolytics, but they do have statistically significant differences in their perception of stress. Healthcare workers in hospital settings have a higher perception of stress than those in outpatient conditions. Three of the six stress factors showed a significant difference between hospital and outpatient conditions. “Workplace organization and financial issues,” “Conflicts and communication at work” and “Professional and intellectual requirements.” All three factors have higher values as stressors in hospital workers. The biggest stress factor in outpatient conditions is “Dangers and harms at work” and “Shift work.” The frequency of use of anxiolytics has nothing to do with variables age, seniority, and occupation. In the variable “Seniority” there was a slight difference, where people with long work experience were more likely to take anxiolytics. Life satisfaction decreases and stress levels increase for hospital health care staff due to working conditions. Outpatient staff did not show an association between working conditions and perceptions of stress or working conditions and life satisfaction, while a weak negative relationship with outpatient staff existed only between the perception of stress and life satisfaction. The obtained results of this research can serve as a starting point for health institutions not only for one county, but of the Croatian health system as a whole and worldwide, to improve the reduction of stress among their workers, but also to raise awareness of the use of anxiolytics and the necessity of caring for mental health, i.e. the promotion of mental health and disease prevention. Satisfaction with the working conditions of health workers at work certainly leads to better health care and an increase in the number of healthy years of life and the quality of life of the community.

**Table 6:** Logistic regression of the possibility of assessing the appearance of factors influencing the use of anxiolytics.

Variable	Log value	SD	Wald	*P*	Odds ratio
Household	-0,53794	0,23129	5,4092	0,0200	0,5840
Perception of stress	0,076283	0,030151	6,4009	0,0114	1,0793
F6 – Professional and intellectual requirement	-0,016050	0,0081785	3,8512	0,0497	0,9841
Help from psychiatrist	−2,78727	0,72628	14,7283	0,0001	0,0616
Regression	4,98993	1,80046	7,6811	0,0056	
Significance level	*P* < 0,0001				
Cox and Snell *R*^2^	0,2106				
Nagelkerke *R*^2^	0,3094				

## Data availability statement

The raw data supporting the conclusions of this article will be made available by the authors, without undue reservation.

## Author contributions

ŽJ: Writing – review & editing, Conceptualization, Supervision, Validation. SP: Formal analysis, Investigation, Writing – original draft. BM: Methodology, Writing – review & editing.
